# Macrocyclic and Hydroxamate Ligands for ^225^Ac Radiopharmaceuticals: Evaluating SSTR2-Targeting Potential

**DOI:** 10.1021/acs.inorgchem.5c04349

**Published:** 2025-12-11

**Authors:** Satoru Tsushima, Ayush Seal, Sergey A. Samsonov, Karim Fahmy, Koichiro Takao

**Affiliations:** † Institute of Resource Ecology, 28414 Helmholtz-Zentrum Dresden-Rossendorf (HZDR), Dresden 01328, Germany; ‡ Laboratory for Zero-Carbon Energy, Institute of Science Tokyo, Ookayama, Meguro, Tokyo 152-8550, Japan; $ Center for Advanced Systems Understanding (CASUS), Görlitz 02826, Germany; § Department of Chemistry, University of Gdańsk, Gdańsk 80-308, Poland

## Abstract

The development of effective radiopharmaceuticals requires careful consideration of the chelator properties and their impact on target receptor interactions. This study comprehensively investigated the molecular interactions between various ^225^Ac radiopharmaceuticals, differing in chelator structure, and somatostatin receptor 2 (SSTR2). The results indicated that 1,4,7,10-tetraazacyclododecane-1,4,7,10-tetra­(methylene)­phosphonic acid (DOTP) represents a promising alternative to the commonly used 1,4,7,10-tetraazacyclododecane-1,4,7,10-tetraacetic acid (DOTA), likely due to its higher negative charge. Incorporation of a poly­(ethylene glycol) linker (PEG4) between the chelator and the peptide moiety of the ligand significantly enhanced receptor activation. Density functional theory calculations identified hydroxamate ligands as potential alternatives to macrocyclic chelators, prompting the synthesis and characterization of a siderophore ligand, N1-[5-(Acetylhydroxyamino)­pentyl]-N26-(5-aminopentyl)-N26,5,16-trihydroxy-4,12,15,23-tetraoxo-5,11,16,22-tetraazahexacosanediamide (DFO*), and its complexation with La^3+^ (a model for Ac^3+^) was confirmed by ^1^H and ^139^La NMR spectroscopy. While DFO* demonstrated high chelating ability and receptor recognition, its flexibility induced significant fluctuations atop the receptor. In contrast, the deoxy variant of the well-studied 3,4,3-LI­(1,2-HOPO) ligand was identified as a structurally suitable chelating agent for ^225^Ac radiopharmaceuticals.

## Graphical abstract

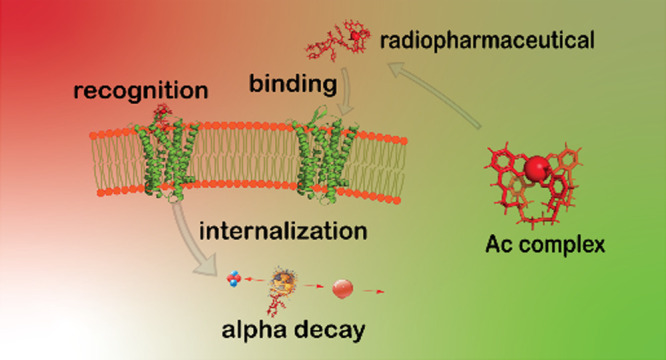

## 1 Introduction

Actinium is a relatively understudied element within the light actinide series, primarily due to the fact that its longest-living isotope has a half-life of only 21.8 years.^1^ This results in a considerable reduction in its availability, which is further compounded by the inherent difficulties in handling it due to its high radioactivity. This, in turn, restricts the scope for undertaking basic studies on actinium chemistry, though several excellent studies have been performed to reveal its coordination chemistry.^1–4^ Its solution chemistry seems relatively straightforward to understand because the +3 oxidation state is virtually the only stable oxidation state of Ac with a 5f^0^ closed-shell electronic configuration, and Ac^3+^ may be considered as a hard acid according to Pearson’s HSAB theory.^5^ This indicates that Ac^3+^ displays affinity for hard bases, including O- and N- donors, in a manner analogous to trivalent lanthanide ions. Over the past decade, research on actinium has increased significantly, with ^225^Ac emerging as a promising candidate for alpha therapy.^6–8^ This was first demonstrated between 2015 and 2016 at the University Hospital Heidelberg (Germany),^9^ where patients with advanced prostate cancer were treated with ^225^Ac-based radiopharmaceuticals, resulting in a significant reduction in prostate-specific antigen levels reaching to undetectable levels.

The principal benefit of utilizing ^225^Ac is that it undergoes four times α-particle emission with an energy of 5.8–8.4 MeV^10^ followed by two times β-decay. This process is similar to the decay of ^227^Ac in the 4*n* + 3 decay chain, also known as the “actinium series”. This process renders it particularly efficacious in the destruction of tumor cells. It is important to note, however, that the daughter isotopes of ^225^Ac exhibit entirely distinct characteristics compared to their parental isotope. For instance, Fr is stable in the +1 oxidation state and has the largest ionic radius of any cations in the periodic table.^11^ It is, therefore, evident that it would be unreasonable to expect a single chelator to function effectively for both ^225^Ac and its daughter ^221^Fr. Following the decay of Ac to Fr, the resulting atom will become a “hot atom” and is unlikely to be retained by the chelator.^12^ It will most likely result in the chemical destruction of the chelator due to the high recoil energy associated with this process. Conversely, it is of great importance to minimize the risk of ^221^Fr and its daughters being released into body fluids following its decay from ^225^Ac. This can be achieved only by ensuring that the ^225^Ac-based radiopharmaceutical is efficiently recognized by the target receptor, thus facilitating its cellular internalization. The objective of this study is thus to optimize Ac-based radiopharmaceutical compounds from both a chemical and a biophysical perspective.

In previous computational investigations, the selection of chelators for Ac^3+^ radiopharmaceuticals was primarily focused on achieving the highest possible affinity. However, ligand recognition of the radiopharmaceutical by the receptor represents a further crucial criterion that must also be taken into account. For this purpose, we conducted a molecular dynamics simulation to examine the structural characteristics and intricate interactions between an Ac^3+^-based radiopharmaceutical and somatostatin receptor 2 (SSTR2). SSTR2 was selected for investigation due to its aberrant expression in numerous cancer cells and tumor blood vessels, as well as its ability to suppress cancer growth and promote cell apoptosis. Accordingly, SSTR2 represents an important drug target for the treatment of various diseases, including neuroendocrine tumors (NETs), thyrotropinoma, and cancer.^13^ The ^177^Lu-labeled somatostatin analogue DOTA-TATE (Lutathera) has also been proven to be an excellent vector for radiation therapy in NETs and has been approved by the U.S. Food and Drug Administration (FDA).^14,15^ Initially, density functional theory (DFT) calculations were utilized to identify suitable Ac^3+^ chelators. Nine chelators were utilized for prescreening based on the DFT calculations, resulting in the selection of four potential candidates: 1,4,7,10-tetraazacyclododecane-1,4,7,10-tetraacetic acid (DOTA), 1,4,7,10-tetraazacyclododecane-1,4,7,10-tetra­(methylene)­phosphonic acid (DOTP), 3,4,3-LI­(1,2-HOPO) (HOPO), and N1-[5-(Acetylhydroxyamino)­pentyl]-N26-(5-aminopentyl)-N26,5,16-trihydroxy-4,12,15,23-tetraoxo-5,11,16,22-tetraazahexacosanediamide (DFO*). The peptide portion of the ligand was fixed as octreotate (TATE). These four chelators and their variants were selected, and ligand molecules were subjected to molecular dynamics simulations in the membrane-bound SSTR2.

## 2 Methodology

### 2.1 Density Functional Theory Calculations

Density functional theory (DFT) calculations were performed on a chelator in the presence or absence of an Ac^3+^ ion using the Gaussian 16 program (Gaussian Inc., Wallingford, CT, USA).^16^ The geometries were optimized in an aqueous phase (eps = 78.3553) using DFT at the B3LYP level with the polarized continuum model (PCM) and the universal force field (UFF) radii. The small core effective core potential (SC-ECP) in conjunction with the corresponding basis sets developed by Dolg et al.^17–19^ was employed on Ac. In the case of H, O, C, and N, an all-electron valence triple-ζ basis set in conjunction with polarization and diffuse functions was employed. The final geometries were confirmed to be energy minima through vibrational frequency analysis, which revealed the absence of any imaginary frequencies. The binding energy was defined as the Gibbs energy difference between the reactants (hydrated Ac^3+^ ion + free chelator) and the products (Ac^3+^-bound chelator + free waters). As DFT was employed in this study for the purpose of prescreening various chelators, entropy correction for “water-in-water” was not implemented, despite the efficacy of this approach in obtaining more accurate reaction energies for systems comprising transition metals and actinides.^20–22^ In this study, we fixed the CN of Ac^3+^ to be 8, except for the hydrated Ac^3+^ ion for which we assumed the CN of 9. The DFT-optimized structure of Ac­(H_2_O)_9_^3+^ ion is given in Figure S1 (SI), where the average distance of Ac and water was estimated to be 2.709 Å, which is slightly larger than the experimental value of 2.63 Å.^3^ However, the discrepancy is at the acceptable level. More details of the DFT calculations are given in the SI.

### 2.2 Materials and Synthesis

DFO*-NH_2_ was prepared as reported elsewhere.^23,24^ The outline of this synthetic procedure is illustrated in Scheme S1 (in the SI) and briefly summarized below. A substitution reaction of 1-bromo-5-chloropentane with potassium phthalimide in DMF afforded *N*-(5-chloropentyl)­phthalimide (yield: 93%), which was further reacted with *tert*-butyl-*N*-benzyloxycarbamate in DMF in the presence of NaI and NaH in a Schlenk flask in an argon atmosphere to give *tert*-butyl *N*-(benzyloxy)-*N*-(5-phthalimidopentyl)­carbamate (yield: 85%). After removal of the phthalimide moiety by using hydrazine monohydrate in EtOH, the primary amine was benzyloxycarbonylated by CbzCl in a water/1,4-dioxane 2:1 mixture in the presence of Na_2_CO_3_ upon cooling on an ice bath, followed by purification with flash chromatography through a silica-gel column with EtOAc/*n*-hexane 1:3 v/v (yield: 85%). Following the Boc-deprotection with trifluoroacetic acid, the product was further reacted with succinic anhydride in pyridine to obtain Bn- and Cbz-protected succinic monoamide (yield: 90%). Its terminal carboxylic group in the succinate moiety was directly condensed with a terminal amino group of DFO through an aid of HATU in the presence of DIPEA and 4-methyl morpholine in DMF, where a mesylate of DFO was employed as a starting material. The residue after solvent evaporation was sonicated in ice-cold acetone (×2) and Milli-Q water (×3). The wet paste was lyophilized to leave a colorless fluffy powder of Bn- and Cbz-protected DFO*-NH_2_ (yield: 23%). Finally, both protecting groups were removed in a H_2_ atmosphere with 10% Pd/C in MeOH, followed by washing with acetonitrile and MTBE and drying in vacuum to give DFO*-NH_2_ (yield: 30%), which was stored at −18 °C in a freezer. The product identity of each synthetic step was confirmed by ^1^H NMR (JEOL ECZL-400, ^1^H: 399.78 MHz).

### 2.3 NMR Spectroscopy (^1^H NMR and ^139^La NMR)

Complexation of DFO*-NH_2_ to La^3+^ was investigated by NMR spectroscopy. The ^1^H NMR spectra of D_2_O–DMSO-*d*_6_ (60:40 v/v) solutions of DFO*-NH_2_ (2.75 mM) with and without LaCl_3_·7H_2_O (2.66 mM) were recorded by a JEOL ECZL-400 (^1^H: 399.78 MHz) at 298 ± 1 K. Triethylamine (19.2 mM) was then injected into the sample solution of DFO*-NH_2_ (2.75 mM) and LaCl_3_·7H_2_O (2.66 mM), and its ^1^H NMR spectrum was acquired as well. Chemical shifts of the obtained NMR spectra were calibrated by the ^1^H resonance of DMSO-*d*_5_, the solvent impurity, at 2.54 ppm. A ^139^La NMR spectrum of the sample solution dissolving DFO*-NH_2_ (2.75 mM), LaCl_3_·7H_2_O (2.66 mM), and triethylamine (19.2 mM) was also accumulated at the resonance frequency of 56.47 MHz.

### 2.4 Molecular Dynamics Simulations

The system setup and parametrization details are provided separately in the Supporting Information. The DFT-optimized structures of the chelators (DOTA, etc.) incorporating Ac^3+^ and a “cap” (which functions as a placeholder) calculated in the previous section were taken and subjected to quantum chemical calculations using the Gaussian 16 program for vibrational frequency analysis as well as the Merz–Kollman scheme^25^ to construct a grid of points around the molecule, constrained by the requirement of reproducing the overall electric dipole moment of the molecule. These Gaussian 16 outputs have been employed to develop force fields for the Ac^3+^-chelator entity using the MCPB.py module,^26^ a Python-based metal center parameter builder utilizing the revised Seminario method^27,28^ in conjunction with the Antechamber module of the AmberTools24.^29^ Atomic partial charges were generated through the two-step restrained electrostatic potential (RESP) method also implemented in the Antechamber module. The missing parameters were further complemented using General Amber Force Field 2 (GAFF2), which generated the complete force field of the Ac^3+^-chelator-cap moieties. The total charge within the cap was constrained to be precisely zero, thereby enabling the cap to be subsequently removed. Finally, the cap was removed from the Ac^3+^-chelator-cap moiety, and the Ac^3+^-chelator entity was manually docked to the peptide N-terminal. The ff19SB force field was assigned to the peptide part, and for D-amino acids (D-Phe and D-Trp), the identical force field parameters developed for their L-configurations were utilized.

The starting structure of SSTR2 (PDB 7T11) was retrieved from the cryogenic electron microscopy structure of SSTR2 from a literature,^30^ in which the receptor was resolved in complex with the octreotide and G-protein. Missing atoms were added by structure refinement. Given the similarity between the peptide portion of the radiopharmaceuticals studied here and the octreotide, the initial position of the ligands in the binding pocket was obtained by the superposition of the peptide portion of the radiopharmaceuticals and the octreotide. Subsequently, the octreotide and G-protein were removed from the system. The PACKMOL-Memgen workflow,^31^ as implemented in the AMBER program package, was employed to embed the adduct of the radiopharmaceutical and receptor into a lipid bilayer comprising 70% phosphatidylcholine and 30% cholesterol. The system was then inserted into a TIP3P water box and neutralized by the addition of K^+^ and Cl^–^ ions to reach a 0.15 M salt concentration. For the assignment of force fields, lipid17 from Dickson et al.^32^ was employed for lipids, and ff19SB for the protein.

Molecular dynamics simulations were conducted using the GROMACS 4.7.0 program package.^33^ Each system was initially subjected to energy minimization through the application of steepest-descent algorithms, without the imposition of positional restraints until the maximum force reached a threshold value of <500 kJ mol^–1^ nm^–1^. Subsequently, NVT and NPT equilibrations were conducted, in which positional restraints were again not introduced, but the temperature was increased in a gradual manner. The NVT equilibration was initiated at *T* = 0 K and was subsequently increased in a gradual manner until *T* = 310 K was reached at the conclusion of the equilibration at *t* = 250 ps (modified Berendsen thermostat with τ_T_ = 0.1 ps). The subsequent NPT equilibration was performed once more, commencing with *T* = 0 K and reaching *T* = 310 K at *t* = 250 ps, using a modified Berendsen thermostat and Parrinello–Rahman barostat. A cutoff of 10 Å, a time step of 2 fs, and the LINCS algorithm^34^ were employed, as well as the particle mesh Ewald method for long-range electrostatics.^35^ The production runs were carried out for 500 ns using the NPT ensemble and a time step of 2 fs. In the case of systems incorporating linkers, it was essential to allow for an adequate equilibrium period for the linker to become fully integrated into the system. For these systems, simulations were conducted for a duration of 550 ns, with the initial 50 ns being excluded from the analysis. Three replicates were generated for each system, resulting in a total simulation time of 1.5 μs.

## 3 Results and Discussion

### 3.1 Comparison of Different Ac^3+^ Chelators

The initial step of this study involved the utilization of DFT calculations to examine the binding affinity of Ac^3+^ in conjunction with a range of chelators. The majority of previous DFT studies employed isolated chelator molecules for this type of investigation. In the present study, a “cap” (−NHCH_3,_ a placeholder) was introduced at either end of the chelator to ensure consistency with the fact that in actual radiopharmaceuticals, the chelator molecule is conjugated to a peptide. In the majority of cases, a cap was introduced via a peptide bond to an existing carboxyl group. However, in some instances, an alternative approach was employed. Figure 1 depicts nine chelators that have been examined along with the introduction of the cap. Their parental complexes are 1,4,7,10-tetraazacyclododecane-1,4,7,10-tetraacetic acid (DOTA, Figure 1A),^36^ its propionic variant (DOTPA, **B**),^37^ diethylenetriaminepentaacetic acid (DTPA, **C**),^38^ 1,4,8,11-tetraazacyclotetradecane-1,4,8,11-tetraacetic acid (TETA, **D**),^37^ 4,7,13,16,21,24-hexaoxa-1,10-diazabicyclo[8.8.8]­hexacosane (2.2.2-cryptand, **E**), ([2-(4,7-bis-carboxymethyl­[1,4,7]­triazacyclononan-1-ylethyl)­carbonylmethylamino] acetic acid) (NETA, **F**),^39^ 1,4,7,10-tetraazacyclododecane-1,4,7,10-tetra­(methylene)­phosphonic acid (DOTP, **G**),^40^ 3,4,3-LI­(1,2-HOPO) (HOPO, **H**),^41^ and N1-[5-(acetylhydroxyamino)­pentyl]-N26-(5-aminopentyl)-N26,5,16-trihydroxy-4,12,15,23-tetraoxo-5,11,16,22-tetraazahexacosanediamide (DFO*, **I**).^42^ Although they have been modified from their parental complexes to include caps, with a sacrifice of functional groups in some cases, for the sake of simplicity, the chelators will henceforth be referred to by the names of their parental complexes (e.g., DOTA). For the initial structures of Ac^3+^-chelator complexes, particularly those of macrocyclic ligands, we took inputs from the existing literature or available crystal structures. Two binding forms for Ac^3+^-macrocycle are known: “encapsulated” and “encircled”. Previous DFT studies have shown that the “encapsulated” form is more stable.^43^ For this reason, this particular conformation has been selected. In the case of the HOPO complex, the crystal structure of the corresponding Eu^3+^ complex^41^ has been taken and Eu^3+^ has been replaced by Ac^3+^. For DFO*, we took a hint from the DFT structure of the Zr^4+^-DFO* complex.^23^

**Figure 1:**
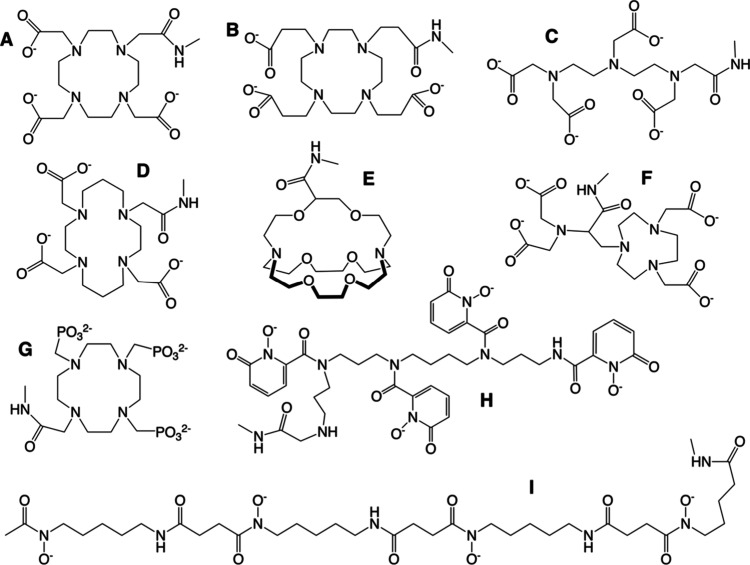
Molecular depictions of the macrocyclic and acyclic amino carboxylate, phosphonate, hydroxypyridonate, and siderophore complexes investigated in this study. Their parental complexes are DOTA (A), DOTPA (B), DTPA (C), TETA (D), 2.2.2-cryptand (E), NETA (F), DOTP (G), HOPO (H), and DFO* (I). See the main text for details regarding complex structures and properties.

Despite the fact that Ac^3+^ is a sizable cation and the ionic radius is slightly larger than that of La^3+^,^44^ an attempt to introduce a chelator with a CN greater than 9 was generally unsuccessful. In this study, we fixed the CN of Ac^3+^ to be 8, except for the aquo complex for which we used [Ac­(H_2_O)_9_]^3+^. In principle, Ac^3+^ exhibits a preference for an 8- or 9-fold coordination,^1^ with DOTA (with CN 8) being a suitable chelator. This result is consistent with previous theoretical investigations^43,45^ and also with the widely held view that DOTA is one of the most optimal chelator for Ac^3+^.^46^ Henceforth, DOTA is regarded as a “gold standard”. Among nine chelators examined, DOTPA (Figure 1B), TETA (**D**), and 2.2.2-cryptand (**E**) exhibit inferior binding affinity relative to DOTA. The calculated binding energies are −91.1, −65.0, −75.3, −14.6 kcal mol^–1^ for DOTA, DOTPA, TETA, and 2.2.2-cryptand, respectively. It was thus resolved that the three chelators inferior to DOTA should be excluded from subsequent investigations. Conversely, DTPA, NETA, HOPO, DOTP, and DFO* are superior Ac^3^^+^-binders, with binding energies of −104.1, −106.3, −104.1, −124.9, and −125.1 kcal mol^–1^, respectively. Notwithstanding the potential error in such calculations, which may amount to 5 kcal mol^–1^ depending on the employed water models (implicit or explicit),^43^ the investigation confirmed these five chelators as stronger Ac^3+^-binders than DOTA, identifying DOTP as one of the most effective Ac^3+^-binders. This is presumably a consequence of a phosphonate group having a larger negative charge (−2) in comparison to a carboxyl group (−1). This point was subjected to rigorous examination in a preceding study by Morgenstern et al.,^43^ and their findings were corroborated in the present investigation. Current findings also suggest that DTPA is a more robust binder than DOTA, likely due to the presence of an additional carboxyl group. It is noteworthy that NETA exhibits superior binding efficacy compared to DOTA, despite the two chelators sharing a comparable number of amine and carboxyl groups. This discrepancy, however, may be attributed to the distinct manner in which the cap has been incorporated into the two chelators (with no sacrifice of a carboxyl group in the case of NETA). Additionally, HOPO was confirmed to be an effective chelator, as previously demonstrated in a study by Ramogida et al.^47^ Among the nine chelators investigated, DFO* was identified as the most effective chelator. Although the coordination environment around Ac^3+^ is similar in the case of HOPO and DFO*, the hydroxamic group in the siderophore has a larger flexibility than that in 1,2-HOPO, despite in both cases forming an Ac–O–N–C-O­(-Ac) chelate ring, thereby favoring DFO*. With regard to HOPO, given that there are four carbonyl oxygens that are not engaged in metal binding, our objective was also to ascertain how their replacement would impact the overall affinity of the chelator for metal binding. This is because we hypothesize that replacing the carbonyl group with −CH_2_ in the backbone will afford increased structural flexibility. From the perspective of density functional theory (DFT) calculations, this replacement resulted in an increase in the binding energy of the Ac^3+^ ion from −104.13 to −106.92 kcal mol^–1^. Although the increase is not substantial, it does indicate that the modified HOPO (deoxyHOPO) is a more effective chelator due to the increase in structural flexibility. Therefore, our subsequent analysis using MD simulations utilizes the deoxy variant of HOPO instead of the conventional one.

We hypothesize here that any chelator exhibiting a binding energy greater than −100 kcal mol^–1^ is strong enough, as this value is greater than that of DOTA. The subsequent analysis using molecular dynamics (MD) simulations will focus on DOTA, DOTP, HOPO, and DFO*, as these four chelators have been demonstrated to be excellent Ac^3+^-binders in both previous and current investigations and also because of a large number of previous studies involving DOTA and HOPO. Chelators such as DTPA or EDTA have been previously proven to be poor chelators due to their instability.^8^

### 3.2 Synthesis of DFO* and Its La^3+^ Binding

In the preceding section, it was determined that DFO* would be the most effective of the chelators listed in Figure 1 and is likely to be the most efficient Ac^3+^-chelator thus far identified. While DOTA, DOTP, and HOPO have all been the focus of extensive research, the binding between An^3+^/Ln^3+^ and DFO* remains unconfirmed through experimental means. Herein, we intend to explore in depth how DFO* interacts with Ac^3+^. NMR spectroscopy is the most promising method for this purpose. However, it is nearly impossible to subject genuine Ac^3+^ to the actual NMR experiments due to specific properties of this artificial radioactive element such as its short half-life (^225^Ac: 9.92 days), strong radioactivity (^225^Ac: 2.17 × 10^15^ Bq·g^–1^), and extreme scarcity. In accordance with this, La^3+^ was selected as an Ac^3+^ surrogate in this study because it is a congener of Ac in the periodic table and actually exhibits the closest ionic radius to Ac^3+^ (Ac^3+^: 1.220 Å extrapolated value, La^3+^: 1.206 Å, CN = 9)^44^ among trivalent metal ions that are experimentally accessible. To verify this hypothesis, we also performed DFT calculations of La^3+^-bound DFO* and aligned the DFT-optimized structure with that obtained for the Ac^3+^-bound counterpart (from the previous section), which confirmed that the two structures look nearly identical (Figure 2c). The diamagnetic nature of La^3+^ with the 4f^0^ electronic configuration is also expedient for the NMR experiments. With regard to the utilization of DFO* in the NMR study, we followed the synthetic procedure outlined by Patra et al.,^23^ wherein the terminal O=CNHCH_3_ moiety (including “cap”) of DFO* (Figure 1, **I**) was replaced by CH_2_NH_2_ or CH_2_NHC­(=O)­C_2_H_4_COOH. Such structural variation from the original DFO* would be less impactful to the binding affinity to metal ions, because the role of this terminal end is for conjugation with a peptide as mentioned above, and the hydroxamic groups would be only utilized to capture metal ions such as Zr^4+^ and Ac^3+^ in accordance with the previous work^23^ or the current DFT calculations (Figure 2c), respectively. For simplicity of the synthetic operations, we have decided to employ the CH_2_NH_2_ variant of DFO* (DFO*-NH_2_) in the actual NMR experiments.

**Figure 2:**
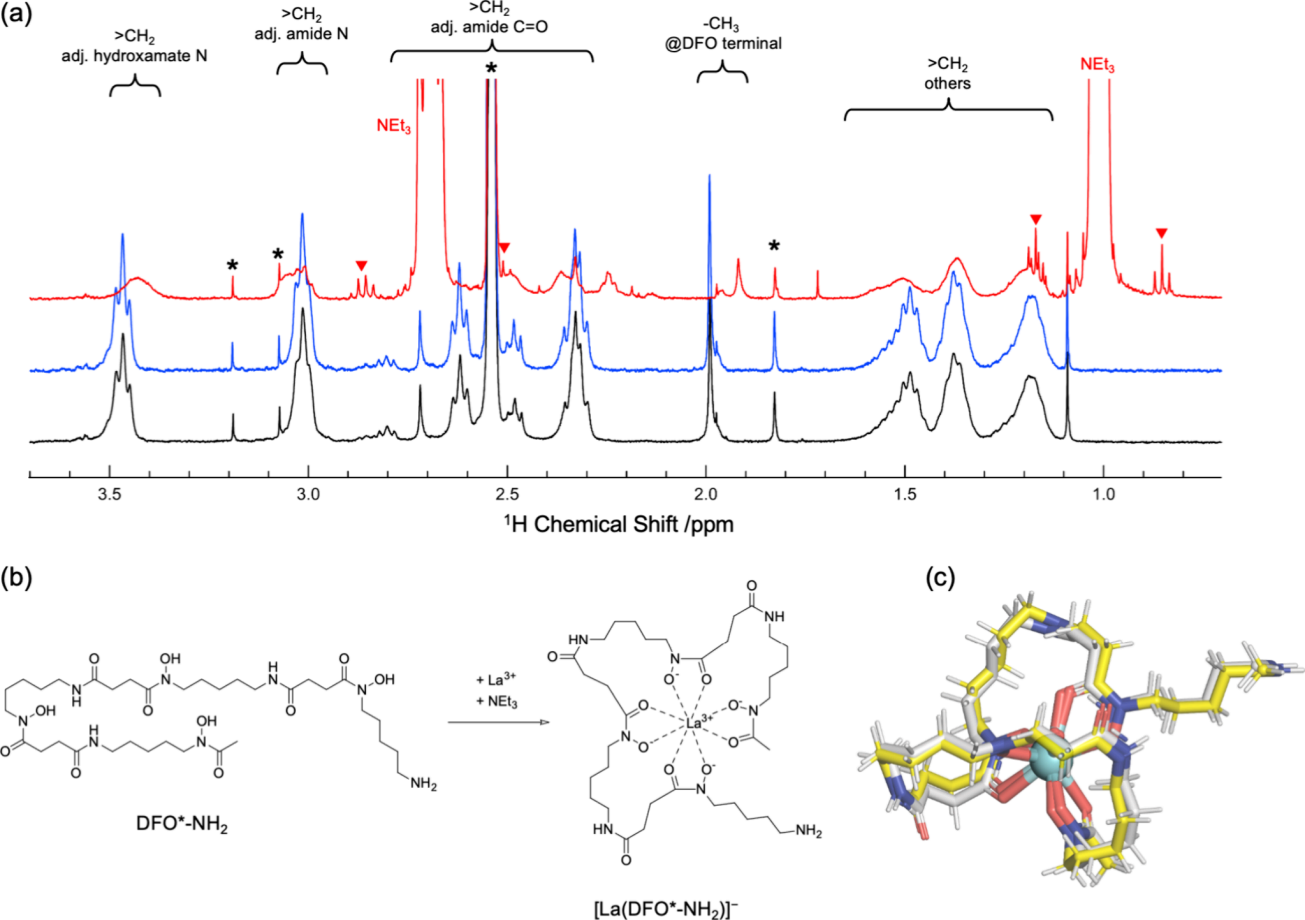
(a) ^1^H NMR spectra of D_2_O/DMSO-*d*_6_ (60:40 v/v) solutions of 2.75 mM DFO*-NH_2_ (black), 2.75 mM DFO*-NH_2_ + 2.66 mM La^3+^ (blue), and 2.75 mM DFO*-NH_2_ + 2.66 mM La^3+^ + 19.2 mM triethylamine (red) at 298 ± 1 K. Asterisk: impurities in solvent or DFO*-NH_2_, Downward triangle: ^13^C satellite peaks of triethylamine. (b) Schematic reaction of La^3+^ capture by DFO*-NH_2_. (c) Superposition of La^3+^-bound DFO* (carbon atoms in gray) and Ac^3+^-bound DFO* (carbon atoms in yellow) obtained by DFT calculations. Ac, N, and O atoms are colored light blue, dark blue, and red, respectively.

Figure 2a shows the variation of the ^1^H NMR spectrum of 2.76 mM DFO*-NH_2_ in D_2_O/DMSO-*d*_6_ (60:40 v/v) upon sequential addition of La^3+^ (2.66 mM) and triethylamine (19.2 mM), where DMSO was needed to fully dissolve DFO*-NH_2_ in a millimolar-level. No significant discrepancies have been observed between the NMR spectra before and after La^3+^ loading. While the actual pH values of such a mixed solvent system are challenging to ascertain, this result is not particularly unexpected under neutral pH conditions, given the weak hydrolytic tendency of La^3+^ (La^3+^ + H_2_O = La­(OH)^3+^ + H^+^, log β_hyd_ = −8.5)^48^ and the p*K*_a_ range of hydroxamates from 8 to 10.^49^ The addition of triethylamine resulted in a shift, broadening, or splitting of the ^1^H signals of DFO*-NH_2_, indicating that its complexation with La^3+^ was assisted by deprotonation of the hydroxamic groups. The observed variation in the NMR spectrum is too complicated for the direct determination of further details regarding the manner in which DFO*-NH_2_ captures La^3+^. However, the eight-coordinated La^3+^ complex of DFO*-NH_2_ is expected to be formed, as illustrated in the product part of Figure 2b in connection with the DFT calculations described above (Figure 2c) and the previously studied Zr^4+^-DFO* system.^23^ Note that 8-fold coordination is commonly observed for both Zr^4+^ and La^3+^ despite differences in their valency. In the case of Pu^4+^ complexation with desferrioxamine E (DFOE), a 9-fold coordination complex with three coordinating waters is formed.^50^ Complexation of DFO*-NH_2_ to La^3+^ was also supported by the silent ^139^La NMR spectrum of this sample solution shown in Figure S2, where the line width of the ^139^La resonance is significantly broadened and undistinguishable from the baseline in an asymmetric environment due to its quadrupolar nature with *I* = 7/2. Finally, we were led to conclude that a stable complex of La^3+^-DFO*, as depicted in Figure 2c, has been successfully formed.

### 3.3 Interactions of Ac^3+^-DOTA-TATE and Ac^3+^-DOTP-TATE with Somatostatin Receptor 2

In the DFT part, the evaluation of chelators was based exclusively on their affinity for Ac^3+^. It is crucial that an Ac^3+^ ion, following its administration into the human body, does not get lost within the body’s fluid pathways en route to the tumor cell. Therefore, it is imperative to preserve the high affinity of the Ac^3+^-chelator and maintain its kinetic inertness. This is essential to preventing Ac^3+^ from undergoing a ligand exchange reaction. Furthermore, the recognition of the radiopharmaceutical by the receptor represents an additional critical criterion that must be considered. The latter aspect has often been overlooked in previous investigations of radiopharmaceuticals. Such an analysis is only possible if a comprehensive molecular representation of the ligand–receptor interaction is available.

In this study, classical molecular dynamics (MD) simulations were employed to model the binding of an Ac^3+^-based radiopharmaceutical with somatostatin receptor 2 (SSTR2), with the aim of elucidating the nature of their interactions. As there are no MD parameters available for an Ac^3+^ ion and also because the Ac^3+^-chelator bond may be considered rigid, we have developed force fields for the Ac^3+^-chelator entity using the MCPB.py,^26^ a Python-based metal center parameter builder utilizing the revised Seminario method^27,28^ in conjunction with the Antechamber module of the Amber program,^29^ as well as quantum chemical calculations using the Gaussian 16 program. In this approach, the bonding and nonbonding parameters involving Ac^3+^ are obtained from density functional theory (DFT) calculations. The full details of the parametrization and calculations are provided in the Supporting Information. The simulated system comprises Ac^3+^-chelator-peptide (referred to here as the “ligand”), SSTR2, lipid bilayers, waters, and ions (K^+^ and Cl^–^). The simulations did not include G-proteins that are adjunct to the receptor for the sake of saving computation time. The construction of the simulated system was carried out using the PACKMOL-Memgen workflow,^31^ as implemented in the AMBER program package.

A simulation of somatostatin receptor (SSTR2) in complex with Ac^3+^-DOTA-TATE was first performed, given the extensive chemical and medical research that has been conducted on this system, as well as its real-world application in the treatment of cancer patients.^51–53^ The cryogenic electron microscopy structure of SSTR2 was taken from a literature,^30^ from which both G proteins and ligand were removed and Ac^3+^-DOTA-TATE as well as missing residues were added. The simulations and subsequent analysis of the MD trajectory (1.5 μs) demonstrated that the ligand (Ac^3+^-DOTA-TATE) exhibited stable binding to SSTR2 throughout the simulation, with an average interaction energy of −964 ± 30 kJ mol^–1^. In average, a total of 26 residues from the receptor were found to be within 3.0 Å distance of the ligand, with more than half (14 residues) being those with hydrophobic side chains. The remaining residues are predominantly those with charged (4) or uncharged polar (6) side chains. This is consistent with the observation that the interaction is largely dominated by Coulomb interactions (−585 ± 57 kJ mol^–1^), though the Lennard-Jones term also amounts to a significant portion (−379 ± 20 kJ mol^–1^). Figure 3 illustrates a representative snapshot from the MD trajectory of the Ac^3+^-DOTA-TATE system. An outward displacement of transmembrane helices 6 (TM6) of SSTR2 at the intracellular side is observed, which is characteristic of the active state of the receptor. Generally, the transition from an inactive to an active form of G protein-coupled receptor (GPCR) involves transitions of TM5, 6, and 7. This comprises an outward displacement of TM5 and 6, an inward movement of TM7 at the intracellular side, and an additional inward shift of TM5 and 7 at the extracellular side.^54^ These parameters may be quantified for SSTR2 based on the work of Lu et al.^55^ on angiotensin II, which belongs to the same class of GPCR as SSTR2, as well as the work by Gervasoni et al.^56^ on SSTR2. In light of the aforementioned considerations, the relevant parameters were calculated on the basis of the MD trajectories, with particular attention paid to (1) the distance between the Cα atoms of residues 218 and 306 and (2) the angle between the Cα atoms of residues 262, 269, and 81. The first quantity accounts for the conformational changes of TM5 and 7 (active <19.0 Å), while the second one reflects the outward movement of TM6 (active >45°). An analysis of the MD trajectory revealed that the active form of Ac^3+^-DOTA-TATE accounted for 23.6% of the total MD frames, which remains at a minor portion.

**Figure 3:**
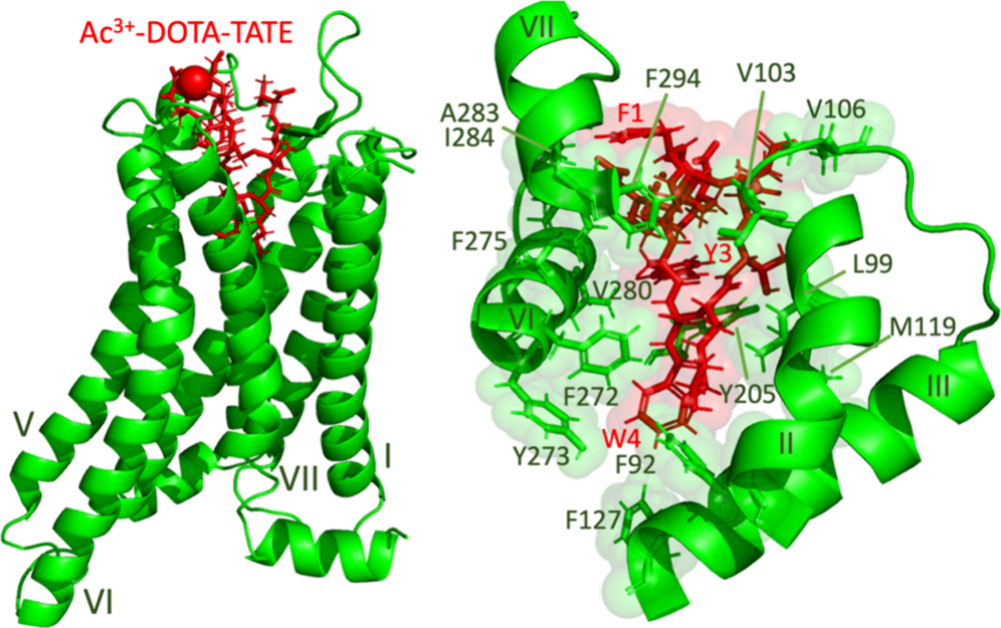
Snapshot of Ac^3+^-DOTA-TATE-bound SSTR2 from an MD trajectory. The ligand (red) and the receptor (green) are shown in a global conformation (left) and a close-up of the binding pocket highlighting hydrophobic interactions (right). SSTR2 transmembrane helices are indicated with Roman numerals. Lipid bilayers, water, and ions are excluded for clarity.

Given that DOTP was identified as a superior Ac^3+^-binder in comparison to DOTA, analogous calculations were conducted on the interaction of Ac^3+^-DOTP-TATE with SSTR2. With regard to the binding energy, the ligand interaction energy with the receptor is −1100 ± 44 kJ mol^–1^. Of this, −753 ± 85 kJ mol^–1^ is accounted for by Coulomb interactions, while −348 ± 22 kJ mol^–1^ is attributed to LJ interactions. The interaction energy for DOTP is clearly greater than that for DOTA (−964 ± 30 kcal mol^–1^ in total), despite considerable fluctuations in the recorded energy values. It can also be observed that the ligand is not as deeply penetrated into the receptor in the case of DOTP. This can be evidenced by a comparison of the distances (distance between the centers of mass of two residues) between Trp^4^ of the ligand and Phe^272^ and Tyr^273^ of the receptor, as well as the distance between Phe^1^ of the ligand and Phe^294^ of the receptor. All of these distances are found to be more than 1.5 Å longer in the case of Ac^3+^-DOTP-TATE compared to Ac^3+^-DOTA-TATE. It would appear that the large negative charge of DOTP is preventing deeper insertion of the ligand into the binding pocket. Conversely, an examination of extracellular loop 2 (ECL2) offers a further perspective on this issue. The root-mean-square fluctuation (RMSF) has been calculated for the Cα atoms of all residues in ECL2, given that ECL2 is the longest loop and is known to play a key role in the interaction with ligand.^57^ The sum of RMSF of the Cα atoms for the DOTA-based system is 34.2 Å, whereas it is 23.6 Å for the DOTP-based system. The fluctuation of ECL2 is prominently decreased in the case of DOTP, which indicates a stronger interaction between ECL2 and the ligand accompanied by a closure of the ECL2 loop. Among the residues in ECL2, Arg^185^ (and Arg^190^) plays a vital role in the closure of the loop through its interaction with the chelator. This is due to its positively charged side chain being in close proximity to the −PO_3_^2–^ group(s) of DOTP. The analysis of the transmembrane helices indicates that the active form accounted for 57.3% of the Ac^3+^-DOTP-TATE trajectory, a substantially larger value compared to that for Ac^3+^-DOTA-TATE (23.6%). Therefore, it can be concluded that the Ac^3+^-DOTP-TATE with (1) higher binding energy, (2) more efficient ECL2 loop closure, and (3) larger activation of the receptor is a much more favorable ligand compared to Ac^3+^-DOTA-TATE.

At this point, our research also encompasses an investigation of the impact of incorporating a linker between the chelator and the peptide. This is a standard practice aimed at ensuring sufficient physical separation between the chelator and the peptide and at minimizing any negative interference between them. In other words, the introduction of a linker is generally thought to minimize any remaining conflicts within the system, given that the chelator and actinium are inherently extraneous in the studied physiological system. In this study, a linker based on poly­(ethylene)­glycols was employed, specifically a PEG_4_-linker as previously studied by Raheem et al.^58^ In general, the type and the length of the linker are significant factors that can influence the efficiency of the ligand, and therefore, their selection is of great importance. However, this paper does not intend to provide a comprehensive analysis of the linker itself. Consequently, our comparison is limited to systems without a linker and those with a PEG_4_ linker. The comparison of the interaction between the ligand and the protein in the absence and presence of the linker indicates the crucial role of the linker in this process. Upon its inclusion, the total interaction energy only slightly changed from −964 ± 30 to −998 ± 22 in the case of DOTA and from −1100 ± 44 to −1098 ± 35 for DOTP (all in kJ mol^–1^ unit). In other words, the overall affinity of the ligand for the receptor is not significantly altered by the inclusion of a linker. However, it is evident that DOTP-PEG_4_ exhibits higher binding energy than DOTA-PEG_4_.

To gain a more detailed understanding, we conducted a more in-depth analysis by examining the contribution from each residue within the ligand. Figure 4 illustrates the interaction between the ligand and the receptor, with the analysis of the ligand presented at the residue level. This approach allows us to identify the critical factors that are essential for the design of a strong binding ligand. The initial focus of our analysis is on the benchmark system, namely, Ac^3+^-DOTA-TATE. The most significant interaction stems from the Coulomb interaction with Lys^5^, which is a positively charged residue. The Lys^5^ plays a significant role in ligand–receptor interactions, irrespective of the nature of the ligand or the presence or absence of the linker. The side chains of Lys^5^ and negatively charged Asp^122^ are in close contact, resulting in robust electrostatic interactions. However, cation-π interactions involving Lys^5^ also play a significant role in the overall interaction landscape, including interactions with Phe^92^ and Phe^272^. Two hydrophobic residues, namely, Tyr^3^ and Trp^4^, also contribute to a notable extent. In general, the “bottom” portion of the ligand plays a more significant role in maintaining the ligand’s binding to the receptor. This trend was also observed in the conformational analysis of the MD trajectory (Figure 3), which revealed intensive hydrophobic interactions between Tyr^3^/Trp^4^ and the receptor, which eventually lead to a conformational change as well as receptor activation. In contrast, the “upper” portion of the ligand contributes less, with the exception of Thr^8^, which interacts prominently with Arg^185^ and Arg^190^ of the ECL2 loop. As previously discussed, however, the ECL2 closure is not a prominent feature in the case of Ac^3+^-DOTA-TATE. The closure becomes more pronounced when Arg interacts directly with the chelator in question, as exemplified by the case of DOTP. This feature, in the case of DOTP, is exclusively associated with its higher negative charge.

**Figure 4:**
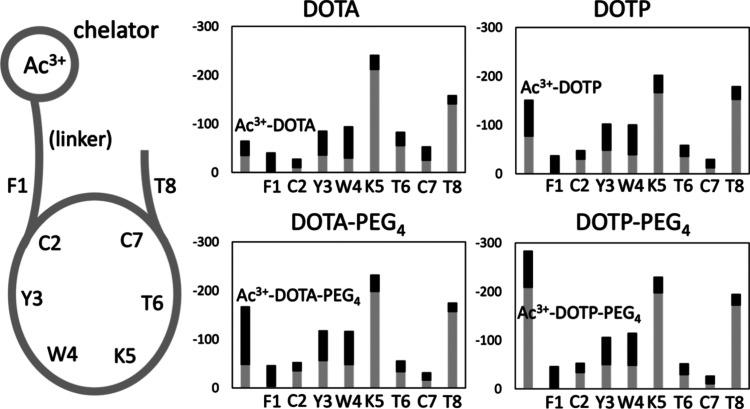
Breakdown of residue-level interaction energies between the Ac^3+^ complexes of DOTA-TATE, DOTP-TATE, DOTA-PEG_4_-TATE, and DOTP-PEG_4_-TATE and somatostatin receptor 2 (SSTR2). Interaction energies were calculated per receptor residue and represent the contribution from Coulombic (gray) and Lennard-Jones (black) interactions expressed in kJ mol^–1^. This analysis details the energetic contributions from the individual residue to the overall binding affinity of each radiopharmaceutical.

Further comparative analysis of the four systems elucidates the function of the linker. A comparison of the respective systems with and without a linker (upper and lower graphs in Figure 4) reveals that the linker plays a beneficial role for both DOTA and DOTP, significantly enhancing the interaction between the chelator (or chelator + linker) part of the ligand and the receptor. Conversely, it is evident that the enhancement of the interaction is predominantly attributed to the Lennard-Jones term in the case of DOTA, whereas it is exclusively attributed to the Coulomb term in the case of DOTP. It thus appears that the function of the linker (PEG_4_ in both cases) exhibits a distinctive profile between DOTA and DOTP. In the system with DOTP without a linker, the Coulomb contribution from the chelator is suppressed, whereas it is significantly liberated when a linker is introduced. This is in contrast to the DOTA system, where the Ac^3+^-DOTA unit is electrically neutral as a whole and there is minimal Coulomb interaction to enhance. In this case, upon introduction of the linker, only a slight additional Coulomb interaction is observed, whereas the system attempts to adapt in order to maximize the interaction in the chelator part.

Finally, we also analyzed the proportion of MD trajectory frames that are in the active form. For DOTA-PEG_4_ and DOTP-PEG_4_, these accounted for 99.7% and 77.7%, respectively, of the total frames in the MD trajectories. These figures are significantly higher than those observed for the corresponding systems without linkers (23.6% and 57.3%, respectively). The calculated parameters collectively indicate that the linker plays a critical role in enhancing the functionality of radiopharmaceuticals. In other words, the incorporation of the linker serves to reinforce the interaction between the ligand and the receptor, thereby augmenting the receptor’s activity. Conversely, the examination of the ECL2 loop revealed a notable elevation (48% and 16% for DOTA and DOTP, respectively) in the RMSF values upon incorporation of the linker. This phenomenon can be attributed to the augmented bulkiness of the ligand resulting from the addition of the linker, which causes the chelator part of the ligand to be partially displaced from the binding pocket. It can be argued that the inclusion of the linker ultimately presents both advantages and disadvantages, and therefore, its size (length) needs to be optimized. However, if receptor activation is efficient, the ECL2 loop closure may not necessarily be a critical factor for the rapid cellular internalization of the radiopharmaceutical. The rise in the RMSF values upon inclusion of the linker in the case of DOTP is a mere 16%, which can be attributed to the pronounced negative charge on the chelator portion of the ligand. This charge exerts a robust interaction with Arg^185^ and Arg^190^ of the ECL2 loop, thereby influencing the loop closure.

### 3.4 Evaluation of the HOPO- and DFO* Based Radiopharmaceuticals

Although DOTP was identified as an excellent alternative to DOTA, our research also encompasses the investigation of an entirely distinct class of ligands, namely, acyclic and hydroxypyridonate, 3,4,3-(LI-1,2-HOPO) (HOPO) and siderophore DFO*, both of which were found to be excellent Ac^3+^ chelators, according to DFT calculations. Given the increase in size of HOPO and DFO* in comparison to two previously studied chelators (DOTA and DOTP), it is reasonable to hypothesize that its interaction landscape may differ significantly. The analysis analogous to the ones we performed on DOTA and DOTP was adopted here to compare the HOPO- and DFO*-based systems with DOTA- and DOTP-based ones.

We first discuss the results of the HOPO system. Analysis of MD trajectory reveals that the interaction energy between the ligand (Ac^3+^-HOPO-TATE) and receptor is −1007 ± 23 kJ mol^–1^, that is, the value between DOTA and DOTP and the fluctuation in energy remains considerably small. The analysis of the active conformation reveals that 88.9% of the frames are in active form, which is a significantly higher value than those for DOTA and DOTP without a linker. It should be noted, however, that the absence of a carboxylic group in HOPO precludes the possibility of connecting the chelator to the peptide in a manner analogous to that achieved with DOTA and DOTP, and a small linker is already implemented in the HOPO system. To ensure compatibility with macrocyclic ligands, we additionally calculated the HOPO system, including the PEG_4_ linker. Furthermore, a deoxy variant of HOPO (deoxyHOPO), as proposed in Section 3.1, was used instead of a standard HOPO. This new system is hence described as Ac^3+^-deoxyHOPO-PEG_4_-TATE. The interaction energy between the ligand and the receptor is calculated to be −1058 ± 22 kJ mol^–1^. Of this, −610 ± 39 kJ mol^–1^ is attributed to Coulomb interactions, while −447 ± 18 kJ mol^–1^ is attributed to LJ interactions. The sum of the interaction energy is similar to that for the DOTP-based system including the PEG_4_ linker, whereas the LJ contribution is much more prominent for the deoxyHOPO-based ligand compared to the DOTP-based system. On the other hand, the fluctuation in energy remains considerably smaller than that for the DOTP system. To gain further insight into the interactions between the ligand and the receptor, a representative snapshot from the MD trajectory is depicted in Figure 5a. In this figure, the ligand and extracellular loop 2 (ECL2) are highlighted in red and blue, respectively. The plot of the ligand position relative to the receptor, as a superposition of 100 MD snapshots, clearly shows that DOTA-PEG_4_ and deoxyHOPO-PEG_4_ systems have the ligand being stably positioned, whereas its position is much more fluctuating in the case of DOTP-PEG_4_ (Figure S3). This observation is consistent with the significantly larger fluctuations in the binding energy observed in the latter system. Furthermore, Figure 5c provides a detailed illustration of the interaction between the chelator and the receptor, highlighting residues within 3 Å from the chelator in green. Upon initial observation, it becomes evident that the interaction is predominantly influenced by the presence of residues with charged or polar side chains with only a single hydrophobic side chain involved. Nevertheless, the decomposition of the interaction between ligands and the receptor for Ac^3+^-deoxyHOPO-PEG_4_-TATE at the residue level (Figure 5b) demonstrates that the Ac^3+^-chelator portion of the ligand exhibits a favorable balance between hydrophilic and hydrophobic interactions. Previous MD simulations on HOPO with a U^4+^ ion^59^ pointed out the valence between hydrophilic and hydrophobic interactions with HOPO being important for having lower conformational flexibility. The inclusion of hydroxypyridinone in a chelator appears to be a good strategy in achieving this strategy.

**Figure 5:**
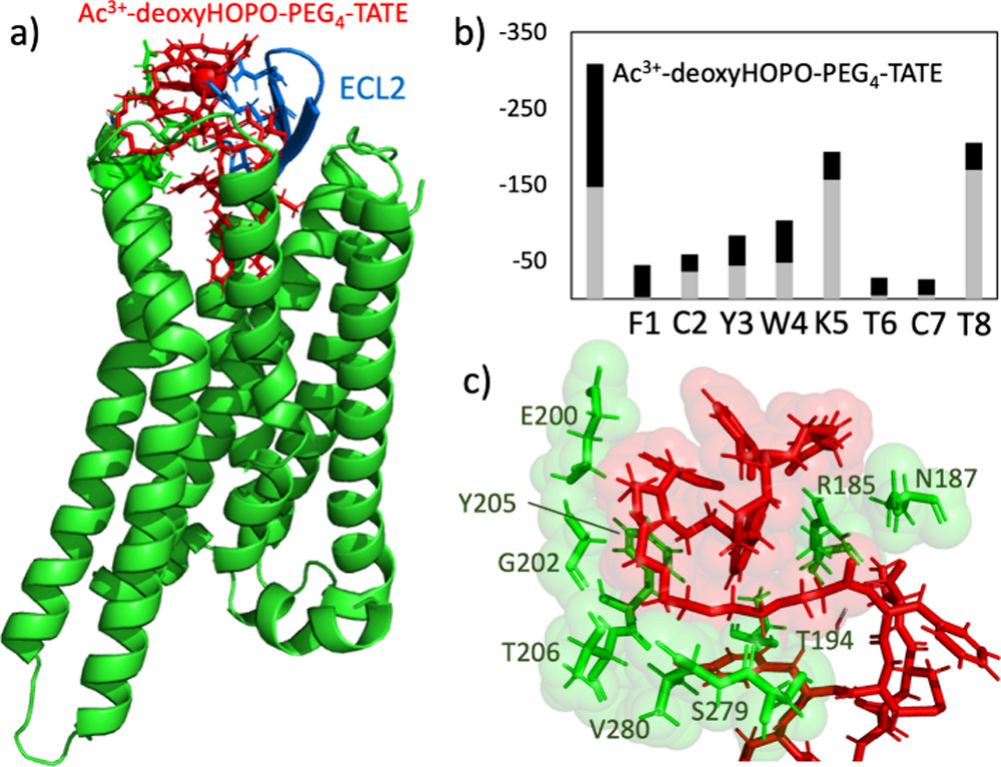
Interaction landscape of the deoxy-HOPO-based radiopharmaceutical. (a) A representative snapshot of Ac^3+^-deoxyHOPO-PEG_4_-TATE-bound SSTR2 from the MD trajectory. The global conformations of the ligand (red) and the receptor (green) are displayed, and the ECL2 loop is highlighted in light blue. (b) The decomposition of the interaction between ligands and the receptor for Ac^3+^-deoxyHOPO-PEG_4_-TATE at the residue level. The gray and black portions represent the Coulomb and Lennard-Jones contributions, respectively (in kJ mol^–1^). (c) The residues in the vicinity (within 3 Å) of the chelator portion of the ligand are highlighted.

In the following, the DFO* system is examined. This system was identified as the most effective among all the chelators in terms of the chelator’s affinity to Ac^3+^, according to DFT calculations. The results of the synthesis and complexation experiments as well as the subsequent NMR analysis have demonstrated that the binding between DFO* and La^3+^ is indeed observed. It is hypothesized that a similar binding interaction may also occur between DFO* and Ac^3+^, and an Ac^3+^-DFO* based radiopharmaceutical was subjected to MD simulations. For this system, the interaction energy between the ligand and the receptor is calculated to be −1052 ± 70 kJ mol^–1^. Of this, −639 ± 92 kJ mol^–1^ is attributed to Coulomb interactions, while −414 ± 38 kJ mol^–1^ is attributed to LJ interactions. It is noteworthy that the interaction energy in this system exhibits significant fluctuations, reaching nearly double the levels observed in other systems. In the DFO* system, the chelator part of the ligand does not firmly sit on top of the receptor and occasionally changes its conformation to a significant extent (Figure S3). A comparison of the DFO* system with the HOPO system reveals that the absence of an aromatic ring in DFO* leads to a greater degree of flexibility in its conformation, leading to enhanced binding affinity for Ac^3+^ relative to HOPO. Conversely, this increased flexibility may also entail certain drawbacks, such as losing the potential rigidity of the chelator portion, which could otherwise facilitate the biofunction of the ligand.

## 4 Conclusions

This study corroborated the efficacy of previously proposed chelators, including DOTA and HOPO, in binding Ac^3+^. The combination of these chelators with TATE (octreotate) results in the creation of an optimal radiopharmaceutical for receptor recognition at somatostatin receptor 2 (SSTR2). The inclusion of the polyethylene glycol linker between the chelator and the peptide moiety of the ligand is also crucial for receptor activation. While DFO* is identified as the strongest Ac^3+^ binder and forms stable complexes with La^3^^+^, it does not appear to be an optimal ligand due to its conformational flexibility. While ligand flexibility has been shown to enhance Ac^3+^ binding affinity in general, this enhancement is counteracted by reduced rigidity, leading to significant fluctuations at the receptor interface. HOPO and its deoxy variant (deoxyHOPO) have been identified as the most promising chelators.

Importantly, this work demonstrates that a chelator’s suitability cannot be determined solely by its affinity for the target metal ion; receptor recognition is also critical. This is of particular importance because ^225^Ac undergoes radioactive decay, transforming into daughter isotopes that may not be retained by the same chelator. Consequently, the recognition of ligands by the receptor, along with the rapid cellular internalization, is of utmost importance. Further investigation is necessary to gain a deeper molecular understanding of the interactions between ^225^Ac radiopharmaceuticals and their target receptors.

## Supplementary Material



## Notes

DFT calculations. A detailed protocol for the system setup of the computational part. Calculated structure of the complex. ^139^La NMR spectra. Superposition of 100 MD snapshots. Synthetic procedure for DFO*-NH_2_ (PDF)
