# Supplementary material for: Macrocyclic and Hydroxamate Ligands for 225Ac Radiopharmaceuticals: Evaluating SSTR2-Targeting Potential
Source: Inorg Chem. 2025 Dec 11;65(14):7592–602. doi: 10.1021/acs.inorgchem.5c04349 (PMC13080970; doi:10.1021/acs.inorgchem.5c04349)

Electronic Supporting Information

Title: Macrocyclic and Hydroxamate Ligands for 225Ac Radiopharmaceuticals: Evaluating SSTR2-Targeting Potential

Authors: Satoru Tsushima,* † ‡ Ayush Seal, †$ Sergey A. Samsonov, § Karim Fahmy, † Koichiro Takao ‡
† Institute of Resource Ecology, Helmholtz-Zentrum Dresden-Rossendorf (HZDR), Dresden 01328, Germany
‡ Laboratory for Zero-Carbon Energy, Institute of Science Tokyo, Ookayama, Meguro, Tokyo 152-8550, Japan
$ Center for Advanced Systems Understanding (CASUS), Görlitz 02826, Germany
§ Department of Chemistry, University of Gdańsk, Gdańsk, 80-308, Poland

*Corresponding author: S.Tsushima (s.tsushima@hzdr.de)

List of Contents

1. DFT calculations
2. Protocol for system setup of computational part
3. Figures
Fig. S1 The structure of the $Ac(H_2O)_9^{3+}$ complex obtained by DFT calculations. The numbers show the Ac-O distances between Ac^{3+} ion and coordinating waters.
Fig. S2 ^{139}La NMR spectra of a D_2O/DMSO-d_6 (60:40 v/v) solution dissolving 2.75 mM DFO*-NH_2 + 2.66 mM La^{3+} + 19.2 mM triethylamine (red) and 10 mM $LaCl_3{\cdot}7H_2O$ in D_2O (black).
Fig. S3 Superposition of 100 MD snapshots (only the ligand and receptor are shown) aligning the structures along the receptor structure for four different systems. Black ribbon, blue lines, and red balls depict the receptor, the ligand, and Ac^{3+} ion, respectively.
4. Scheme
Scheme S1 Synthetic procedure for the synthesis of DFO*-NH_2.
----
1. DFT calculations

The default integration grid was selected, which is UltraFine pruned grids (99 radial shells, 590 angular points per shell) for all atoms except for Ac, for which unpruned grid (199, 590) has been used. Default geometry optimization setting was used, where threshold values for convergence is maximum and R.M.S. of the force of 0.00045 a.u. and 0.00030 a.u., respectively, and maximum and R.M.S. displacements of 0.0018 a.u. and 0.0012 a.u., respectively.

2. Protocol for system setup of computational part

Step 1 Gaussian16 geometry optimization
Make a Gaussian input of a molecule consisting of Ac and chelator with a cap and save it as "MOL_cap.gjf". Run a geometry optimization job in solvent.

Step 2 Gaussian16 vibrational frequency job
Take the optimized structure of Step 1 and run frequency job "MOL_cap_fr.gjf". Save also the chk file (by including %chk=MOL_cap.chk line in the input).

Step 3 Gaussian16 Electrostatic Potential
Take the optimized structure of Step 1 and produce charges fit to the electrostatic potential at points selected according to the Merz-Singh-Kollman scheme "MOL_cap_mk.gjf". The Gaussian input includes “Pop(MK, ReadRadii) IOp(6/33=2,6/42=6)” with “Ac 1.739” at the end of the input.

Step 4 Run antechamber of AmberTool to generate mol2 and pdb files

```
% antechamber -o MOL_cap.mol2 -fo mol2 -c resp -fi gout -i MOL_cap_mk.log -s 2 -dr no
% cp ANTECHAMBER.ESP MOL.esp
```

% cp ANTECHAMBER_RESP1.IN MOL-step1.respin

Edit "MOL-step1.respin" to put charge constraints such as freezing charges of all atoms except for the cap and its vicinity, fix the total charge of the cap to be exactly 0.0000. Set &cntrl parameters; nmol = 1, ihfree = 0, iqopt = 2, ioutopt = 0, qwt = 0.0005.

% resp -O -i MOL-step1.respin -o MOL-step1.respout -e MOL.esp -t MOL-step1.crg -q QOUT

Take charges from "MOL-step1.crg" and manually overwrite those in "MOL_cap.mol2". Edit "MOL_cap.mol2" also to add bonds between metal and chelator (in DOTA example, four Ac-O and four Ac-N bonds)

% antechamber -o HOPO3_cap.pdb -fo pdb -fi mol2 -i HOPO3_cap.mol2 -dr no

Step 5 Divide mol2 files into Ac part and chelator part
% cp MOL_cap.mol2 AC.mol2
Edit and leave only Ac
% cp MOL_cap.mol2 MOL.mol2
Edit and leave only chelator+cap

Nomenclature must be carefully edited for "MOL_cap.pdb", "AC.mol2, "MOL.mol2"

Step 6 Generate force constants using Seminario method
% MCPB.py -i MOL_cap.in -s 1
% MCPB.py -i MOL_cap.in -s 2 --fchk=MOL_cap.fchk
(fchk file from Gaussian16 frequency job)

Below is an example of the file "MOL_cap.in"

```
original_pdb MOL_cap.pdb
group_name MOL
cut_off 2.8
ion_ids 1
ion_mol2files AC.mol2
naa_mol2files MOL.mol2
```

% cp MOL_mcpbpy.frcmod MOL_cap.frcmod

Step 7 Construction of the "real" ligand system
Using Gaussian16 output from Step 1 and also using the pdb file of peptide part of radiopharmaceutical, construct manually a PDB file which include Ac, chelator, and peptide "MOL_cap+pep.pdb"

Step 8 Assigning parameters on the ligand part

% xleap -sf xleap_first.in

Below is an example of the file "xleap_first.in"

```
source XXXX (here insert relevant path/name)
source XXXX (here insert relevant path/name)
loadamberparams XXXX (here insert relevant link/name)
loadamberparams MOL_cap.frcmod
MOL = loadmol2 MOL_cap.mol2
list
pep =loadpdb MOL_cap+pep.pdb
```

on the menu of xleap

% edit pep

You will see that both chelator and the first residue have extra atoms automatically added by xleap. Remove those added atoms from both and add a bond between chelator and peptide and add also add S-S bond if necessary.

% saveoff pep pep.lib

Edit "pep.lib" manually and rename residues if necessary (from “NPHE” to “PHE”, for instance), rename Ac and N/O (to M1, Y1, etc, to be consistent with the naming in “MOL_cap.frcmod”), and few atoms in the very first residue may need to be renamed as well as charge reassigned (to that of PHE, and not that of NPHE, for instance).

% xleap -sf xleap_second.in

(For “xleap_second.in”, add “loadoff pep.lib” at the last line of “xleap_first.in”)

on the menu of xleap

% edit pep
% charge pep
(Make sure that the total charge is integer. If not there is something wrong in the above procedure.)
% check pep

Missing parameters will be displayed. Manually add missing parameters to "MOL_cap.frcmod" by taking same/similar bonding parameters from GAFF (general AMBER force field). Quit xleap and restart "xleap -sf xleap_second.in" and repeat the same procedure until no missing parameter is detected.

Step 9 Construction of the ligand + receptor
Manually combine ligand and receptor (the pdb file of the receptor needs to be separately prepared with missing atoms added and the protonation state adequately corrected) on (for instance) PyMOL and save the docked molecule in PDB format as "aligned.pdb"

Step 10 Insertion of ligand+receptor into lipid bilayer
Insert ligand+receptor (“aligned.pdb”) in POPC 70% + Cholesterol 30% lipid bilayer using PACKMOL-memgen.

% packmol-memgen --pdb aligned.pdb --lipids POPC:CHL1 --ratio 7:3 --salt --salt_c K+ --saltcon 0.15 --keepligs --notprotonate --nottrim

Step 11Reconcile .lib and .pdb coordinates
Divide the produced pdb file of the entire system into two files; ligand only and the rest. Edit the coordinate of atoms in "pep.lib" to have exactly the same coordinates with the ligand part of the pdb.

Step 12
% tleap -sf tleap2.in
(Check if the charge of the box is neutral. If not, delete Na+ or Cl- and redo the previous step.)

% sander -O -i init_min.in -o lrl_min1.out -p lrl.prmtop -c lrl.inpcrd -r lrl_init1.rst -ref lrl.inpcrd
% sander -O -i init_min2.in -o lrl_min2.out -p lrl.prmtop -c lrl_init1.rst -r lrl_init2.rst
(Test runs in order to check if everything is fine)

% cpptraj -p lrl.prmtop -y lrl_init2.rst -x lrl_init2.pdb
(Get geometry after initial run in PDB format)

% acpype -p lrl.prmtop -x lrl.inpcrd
(Conversion to Gromacs inputs)

3. Figures

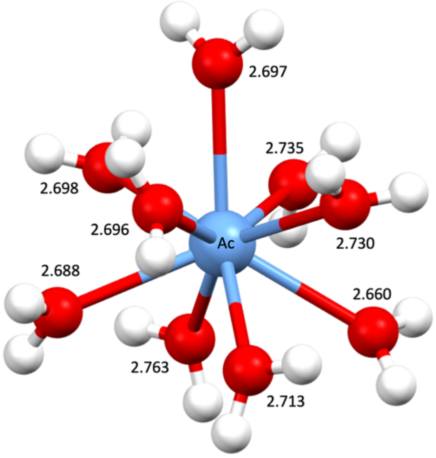

Fig. S1 The structure of the $Ac(H_2O)_9^{3+}$ complex obtained by DFT calculations. The numbers show the Ac-O distances between Ac^{3+} ion and coordinating waters (unit in Å).

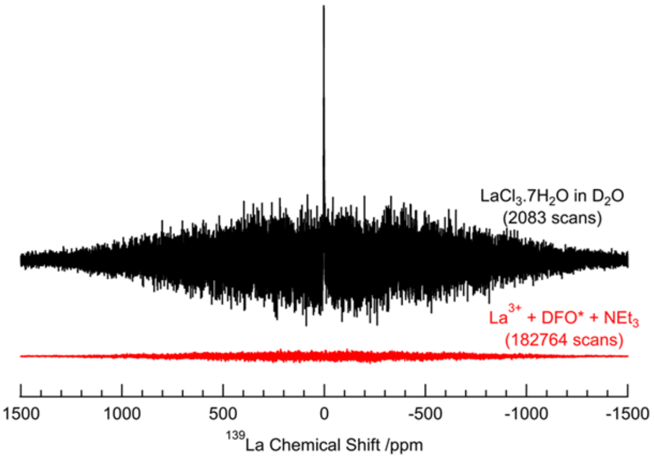

Fig. S2 ^{139}La NMR spectra of a D_2O/DMSO-d_6 (60:40 v/v) solution dissolving 2.75 mM DFO*-NH_2 + 2.66 mM La^{3+} + 19.2 mM triethylamine (red) and 10 mM $LaCl_3 \cdot 7H_2O$ in D_2O (black).

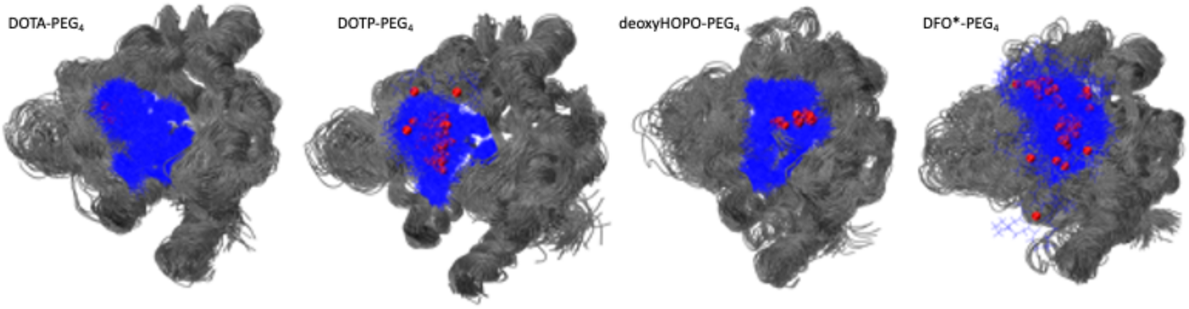

Fig. S3 Superposition of 100 MD snapshots (only the ligand and receptor are shown) aligning the structures along the receptor structure for four different systems. Black ribbon, blue lines, and red balls depict the receptor, the ligand, and Ac^{3+} ion, respectively.

4. Scheme

Scheme S1 Synthetic procedure for the synthesis of DFO*-NH_2.

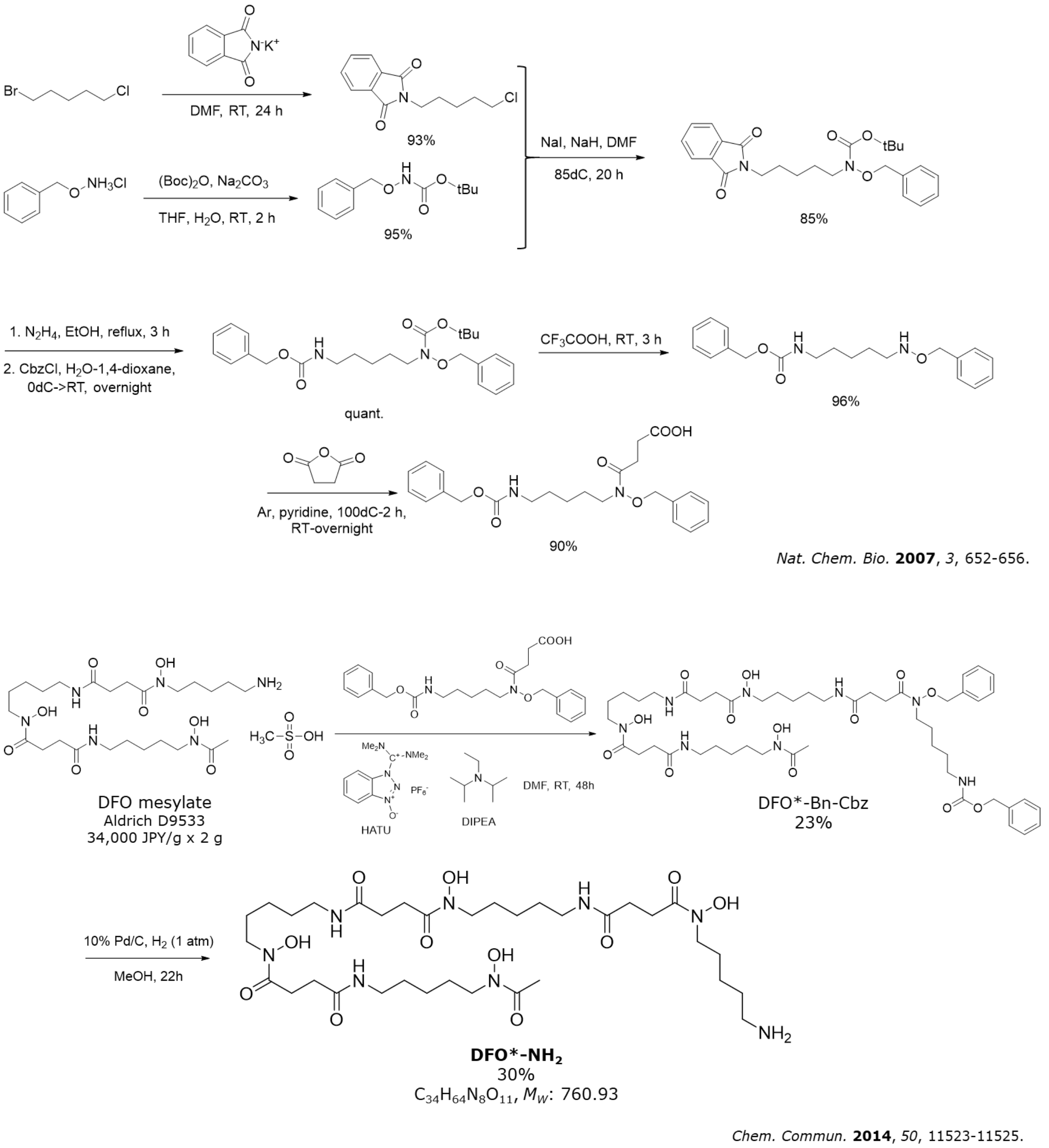

Supplement: Supplementary file 1 [file ic5c04349_si_001.pdf]
